# Prognostic value of epicardial and pericardial adipose tissue in patients with chronic heart failure

**DOI:** 10.1007/s12471-026-02077-z

**Published:** 2026-09-16

**Authors:** Yoran Crum, Dirk J. van Veldhuisen, Michelle Lobeek, Adriaan A. Voors, Michiel Rienstra, Thomas M. Gorter

**Affiliations:** University Medical Centre Groningen, Department of Cardiology, University of Groningen, Hanzeplein 1, Groningen, The Netherlands

**Keywords:** Anthropometric measure, Epicardial adipose tissue, Fat distribution, Heart Failure, Obesity, Pericardial adipose tissue, Prognostic marker, Echocardiography

## Abstract

**Background:**

Central obesity, rather than overall obesity, may influence heart failure (HF) outcomes. Alternative anthropometric measures remain limited in assessing the actual amount and distribution of visceral adiposity. We hypothesize that epicardial and pericardial adipose tissue (EAT and PAT) are easily obtainable on standard echocardiography and provide prognostic value in patients with HF.

**Methods:**

In total, 572 patients with HF with reduced, mildly reduced and preserved ejection fraction (HFrEF, HFmrEF, and HFpEF, respectively) were included. EAT (the adipose tissue between the myocardium and the visceral layer of the pericardium) and PAT (the adipose tissue outside the parietal layer of the pericardium) thickness were measured on echocardiography and expressed in mm. Patients were followed for the combined outcome of all-cause mortality and HF hospitalization.

**Results:**

Mean age was 67 ± 14 years, 38% were women, 346 had HFrEF, 100 had HFmrEF and 126 had HFpEF. Mean body mass index (BMI) was 27 ± 6 kg/m^2^, mean EAT thickness 4.8 ± 2.1 mm, and mean PAT thickness 5.7 ± 2.8 mm. During a median follow-up of 3.2 ± 1.9 years, both EAT and PAT thickness were associated with an increased risk for the combined outcome [HR 1.15 (95% CI 1.03–1.30), *p* = 0.006 and HR 1.25 (95% CI 1.10–1.41), *p* < 0.001, respectively], whereas BMI was not. In a multivariable model, only PAT thickness remained associated with poor outcome [HR 1.49 per SD (95% CI 1.10–2.03), *p* = 0.011].

**Conclusion:**

Increased PAT thickness, more than EAT, assessed on routine echocardiography, may serve as a valuable prognostic marker in chronic HF.

**Supplementary Information:**

The online version of this article (https://doi.org/10.1007/s12471-026-02077-z) contains supplementary material, which is available to authorized users.

## What’s new?

Previous studies have primarily focused on the role of general obesity in patients with heart failure (HF), whereas obesity is defined according to body mass index (BMI). However, BMI is limited in assessing the actual burden of adipose tissue (AT). In addition, alternative anthropometric measures, such as waist circumference and waist-to-height ratio, also remain limited in assessing the amount and distribution of AT. Fat depots of increasing interest are epicardial and pericardial adipose tissue (EAT and PAT, respectively), which are situated close to the heart. In the present, large study population, we demonstrated that EAT and PAT measured on routine echocardiography are associated with poor outcome, irrespective of overall obesity. These specific AT depots may thus serve as easily obtainable prognostic markers in patients with HF, irrespective of overall obesity.

## Introduction

Obesity has been repeatedly shown to be an important risk factor for the development of heart failure (HF), but the prognostic value of overall obesity in established HF remains disputed [1, 2]. The association between obesity and HF has been generally based on increased body mass index, which is limited in assessing the burden of adipose tissue [3]. Furthermore, alternative anthropometric indices of central adiposity, such as waist circumference and waist-to-height ratio, have been associated with poor outcomes [4, 5]. Nevertheless, these alternative indices are also limited in assessing the actual amount and distribution of adipose tissue (AT) [6].

**Fig. 1:**
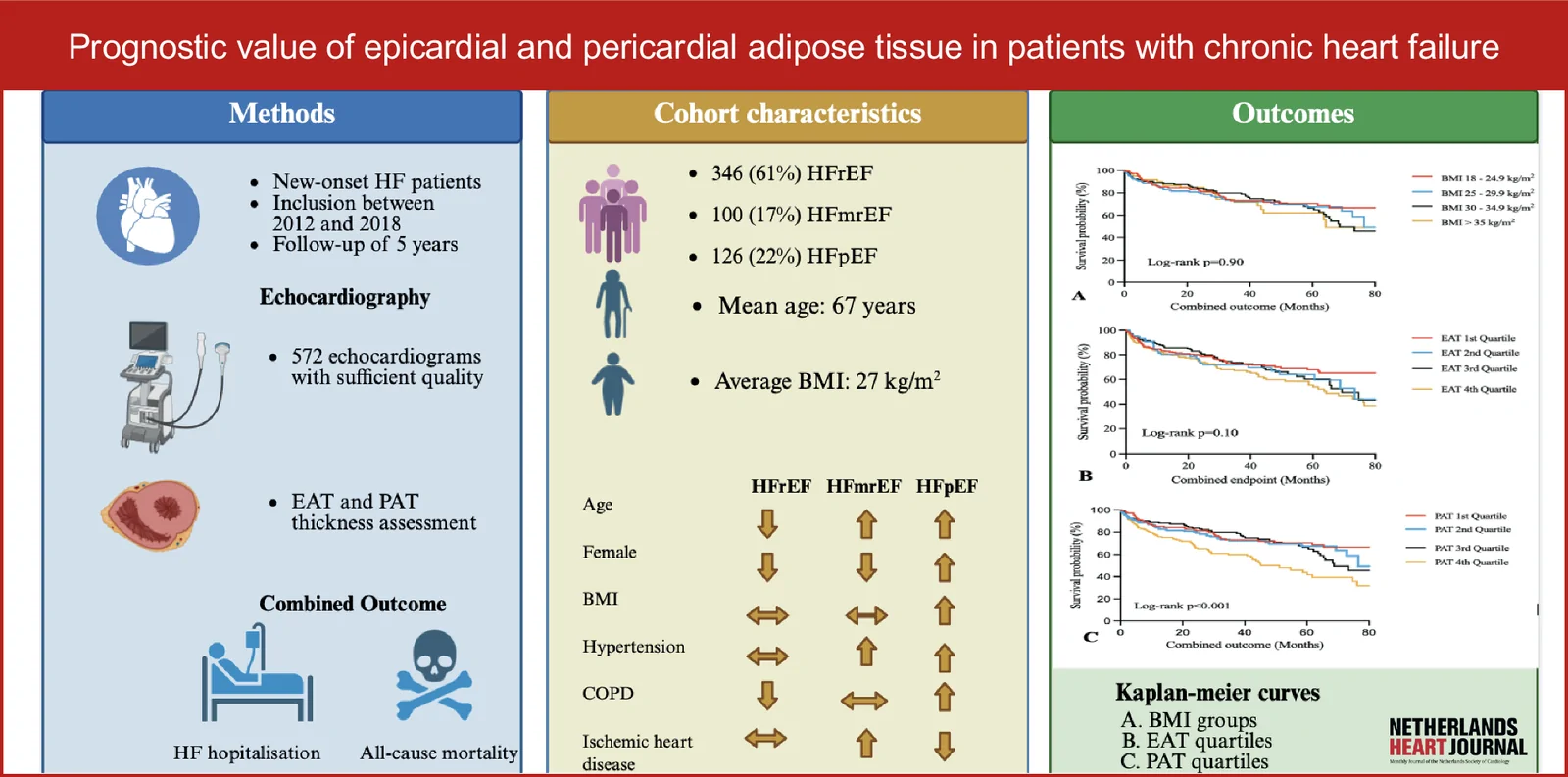
Infographic

Specifically, AT depots in proximity to the heart (e.g., epicardial and pericardial adipose tissue [EAT and PAT]) may have clinical relevance in patients with HF [7]. EAT is located within the visceral layer of the pericardium and directly adjacent to the myocardium and the coronary arteries. Recent studies have shown that EAT is associated with disease severity, HF hospitalization, and mortality in patients with HF [8, 9]. In contrast, less is known about PAT in patients with HF, which differs in anatomical location, embryological origin, and blood supply. PAT may represent a more overall unhealthy cardiometabolic profile (Fig. 1; [6, 10]).

While EAT and PAT have both been suggested to be involved in the development of HF, the association of these very distinct fat depots with clinical outcomes remains unclear. Therefore, we aimed to assess the association between EAT and PAT measured on standard echocardiography with clinical outcomes in patients with established HF. We also investigated whether these AT depots show a differential association with outcomes in patients with HF with reduced versus mildly reduced and preserved ejection fraction.

## Methods

All patients with new-onset HF included in the present study originated from a prospective observational cohort that has been described previously in detail [11, 12].

In brief, new-onset HF patients were referred to the specialized HF outpatient clinic of our tertiary care hospital between 2012 and 2018. Patients were subsequently classified as having HF with reduced ejection fraction (HFrEF), with mildly reduced ejection fraction (HFmrEF), and with preserved ejection fraction (HFpEF), according to the left ventricular ejection fraction (LVEF). HF had been diagnosed by a cardiologist no more than 3 months before inclusion, according to the European Society of Cardiology Guidelines for HF [13]. Patient characteristics were recorded at baseline visits [12]. Participants were categorized into four BMI categories: 1) BMI < 25 kg/m^2^; 2) BMI 25–29.9 kg/m^2^; 3) BMI 30–34.9 kg/m^2^; and 4) BMI ≥ 35 kg/m^2^.

The study complied with local and national regulations regarding retrospective clinical research, as confirmed by the medical ethical evaluation committee, and was conducted in accordance with the principles of the Declaration of Helsinki.

### Outcome assessment

The primary outcome measure was time to the first event of either HF hospitalization or all-cause mortality. Outcome data were extracted from the hospital’s electronic health record until the censor date of 1 September 2018.

### Echocardiography

Echocardiography was performed as part of routine clinical care. All echocardiograms were digitally stored and transferred to the Echo Core Lab for additional measurements using EchoPAC software (Version B12).

**Fig. 2:**
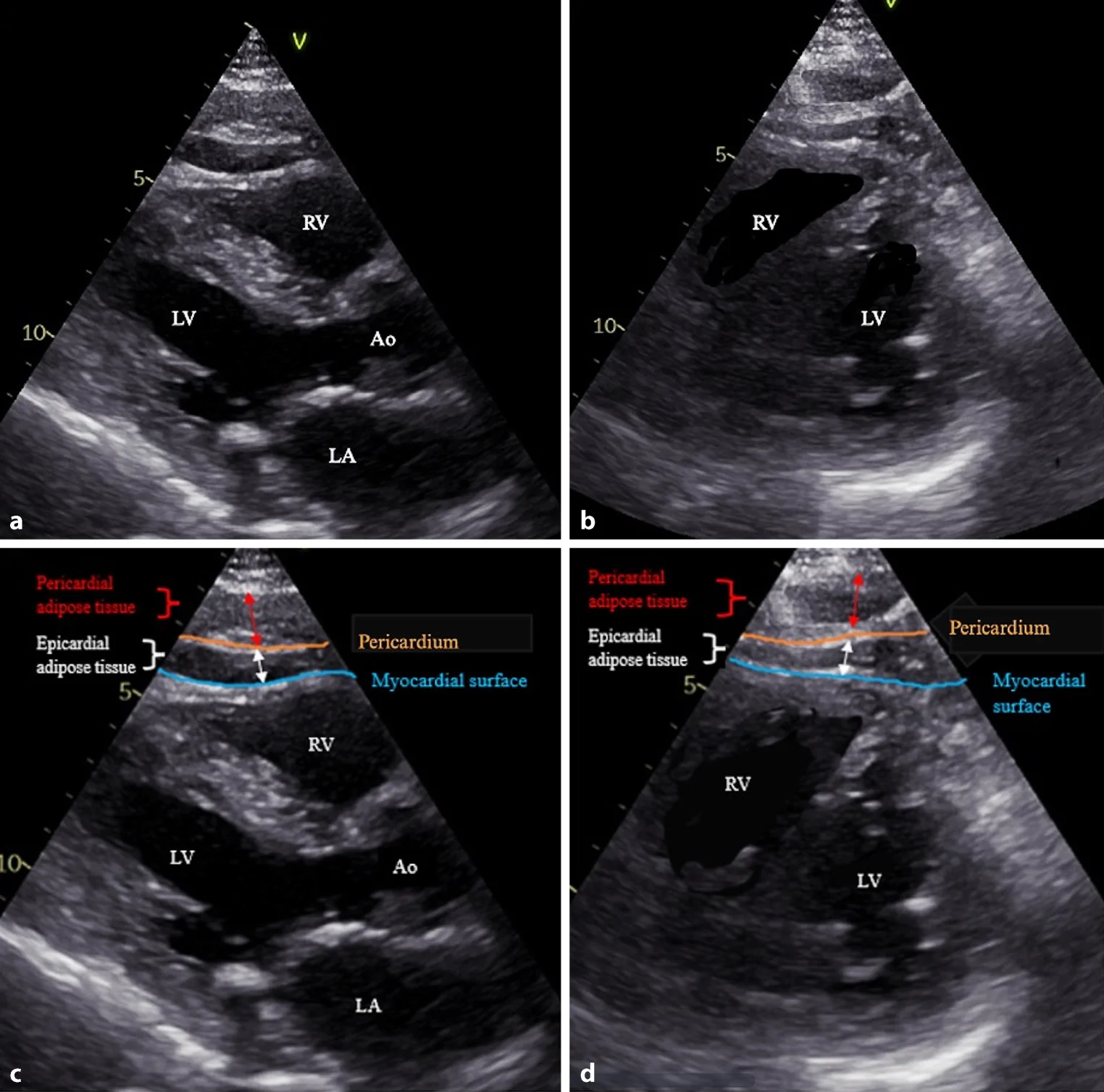
Location of epicardial- and pericardial tissue depots and echocardiographic assessment. **a**, **b** Show the parasternal long-axis and short-axis views on the echocardiogram. **c**, **d** Show the different layers of adipose tissue (i.e. EAT and PAT) surrounding the myocardium. *White*: EAT, *Red*: PAT. Using the aortic annulus and interventricular septum as reference points. The *orange line* indicates the pericardium, and *blue line* indicates the myocardial surface.* Ao* Aorta;* EAT* epicardial adipose tissue;* PAT* pericardial adipose tissue;* LA* left atrium;* LV* left ventricle;* RV* right ventricle

In total, 60 echocardiograms were excluded due to insufficient image quality. EAT and PAT thickness were measured alongside the right ventricle (RV) of the parasternal short-axis and parasternal long-axis views, as illustrated in Fig. 2. The measurements were obtained over two cardiac cycles at the end of systole and were subsequently averaged. The papillary muscles/interventricular septum and aortic annulus were used as anatomic reference points for these assessments [14]. PAT was defined as the echolucent area outside the pericardium, in which the visceral and parietal layers can be seen sliding over each other on the echocardiographic image loops, facilitating a clear distinction between EAT and PAT. Patients were divided into quartiles according to EAT and PAT thickness. All EAT and PAT measurements were performed by one experienced investigator (Y.C.). For interobserver variability, > 10% of the measurements were independently re-evaluated by another experienced investigator (T.M.G.), demonstrating differences of ≤ 5% between measurements. Intra-observer variability was likewise ≤ 5%.

### Statistical analysis

Data were presented as mean ± standard deviation, median [interquartile range], or numbers (%). Differences for continuous variables between two groups were tested using the Independent samples t‑test or Mann-Whitney U test, according to distribution. The chi-square test was used for categorical variables. Intergroup differences were tested using one-way ANOVA or the Kruskal-Wallis test. Post hoc Bonferroni correction was applied to adjust for multiple comparisons. Correlations between continuous variables were tested using Pearson correlation analysis. Hazard ratios (HR) for continuous variables are depicted as per standard deviation with 95% confidence interval (CI). Cox proportional hazards regression analyses were performed to assess the prognostic value of EAT and PAT, adjusting for confounders identified from previous literature and variables associated with outcome in univariable analysis (*p* < 0.05), using a backward selection approach [15]. For EAT and PAT, spline curves were plotted up to the 95th percentile of the distribution to minimize the impact of outliers. Associations between outcomes and EAT, PAT, and BMI groups were assessed using log-rank tests. For all statistical analyses, a two-tailed *p* < 0.05 was considered statistically significant. SPSS Statistics 27 version (IBM SPSS Statistics, NY, USA) was used to conduct statistical analyses, and spline curves were constructed using R.

## Results

In total, 572 patients with HF and available LVEF assessment had echocardiograms of sufficient quality for EAT and PAT measurements. The study population consisted of 346 (61%) patients with HFrEF, 100 (17%) with HFmrEF, and 126 (22%) with HFpEF. Most patients were men (*n* = 355, 62%). As shown in Tab. 1, patients with HFpEF and HFmrEF were older, more frequently had atrial fibrillation and hypertension, had a higher BMI, and had lower NT-proBNP levels compared with patients with HFrEF.

**Table 1:** Baseline Characteristics

	Total (*n* = 572)	HFrEF (*n* = 346)	HFmrEF (*n* = 100)	HFpEF (*n* = 126)	*P* for trend
Age, years	67 ± 14	66 ± 14	69 ± 15^§^	70 ± 14^§^	**0.003**
Sex, women %	217 (38%)	131 (35%)	35 (33%)	51 (52%) ^†^	**0.002**
Body mass index (kg/m^2^)	27 ± 6	27 ± 5	27 ± 5	29 ± 7^§†^	**< 0.001**
*HF characteristics*					
NYHA Class					0.4
I	182 (30%)	53 (14%)	20 (19%)	17 (13%)	
II	325 (52%)	203 (54%)	56 (52%)	63 (46%)	
III	100 (16%)	107 (29%)	29 (27%)	51 (38%)	
IV	7 (1%)	6 (2%)	1 (1%)	3 (2%)	
*Comorbidities*					
Hypertension	216 (34%)	114 (31%)	44 (41%)	56 (41%)	**0.027**
Ischaemic heart disease	237 (38%)	143 (38%)	49 (45%)	43 (32%)^†^	0.089
Atrial fibrillation, history	241 (38%)	130 (35%)	39 (36%)	66 (49%)^§†^	**0.016**
Diabetes mellitus	138 (22%)	72 (19%)	27 (25%)	38 (28%)	0.083
COPD	85 (14%)	35 (9%)	17 (16%)	31 (23%) ^§^	**< 0.001**
Cardiothoracic surgery	94 (15%)	46 (12%)	22 (20%)	25 (18%)	0.055
*Laboratory tests*					
eGFR, ml/min/1.73 m2	67 ± 25	67 ± 25	69 ± 26	64 ± 26	0.4
NT-proBNP, ng/l	1538 [558–3679]	1637 [693–3770]	1013 [359–2121]^§^	963 [359–2037]^§^	**< 0.001**
*Medication*					
Beta-blocker use	534 (85%)	333 (89%)	90 (83%)	105 (77%)^§^	**0.003**
ACEi/ARB use	521 (83%)	325 (87%)	91 (84%)	101 (74%)^§^	**0.003**
Loop diuretic use	437 (70%)	261 (70%)	72 (67%)	99 (73%)	0.6
*Echocardiographic parameters*					
LVEF (%)	38 ± 13	28 ± 8	45 ± 3^§^	55 ± 4^§†^	**< 0.001**
LAVI	43 ± 15	44 ± 16	42 ± 12	43 ± 15	0.5
LVMI	107 ± 32	111 ± 32	102 ± 28^§^	100 ± 34^§^	**0.002**
e′ lateral	8.3 ± 3.2	7.7 ± 3.2	8.7 ± 3.1	9.1 ± 3.3^§^	**< 0.001**
e′ septal	5.9 ± 2.3	5.5 ± 2.1	6.2 ± 2.3^§^	6.7 ± 2.5^§^	**< 0.001**
e′ mean	7.1 ± 2.5	6.7 ± 2.4	7.4 ± 2.2	8.0 ± 2.6^§^	**< 0.001**
E/e′	12 ± 5	12 ± 6	11 ± 4	12 ± 5	0.4
EAT	4.8 ± 2.1	4.5 ± 2.1	4.9 ± 1.8	5.4 ± 2.1^§^	**< 0.001**
PAT	5.7 ± 2.8	5.4 ± 2.4	5.5 ± 2.9	6.4 ± 3.5^§†^	**0.004**

Data are mean ± SD, median [interquartile range] or *n* (%). Final column reflects overall differences in EAT tertiles. ANOVA was used for normally distributed parameters and Chi-squared was used for categorical parameters

*ACEi* angiotensin-converting enzyme inhibitor; *ARB* angiotensin receptor blocker; *BMI* body mass index; *COPD* chronic obstructive pulmonary disease; *DBP* diastolic blood pressure; *eGFR* estimated glomerular filtration rate; *HF* heart failure; *HFrEF* heart failure with reduced ejection fraction; *HFmrEF* heart failure with mildly reduced ejection fraction; *HFpEF* heart failure with preserved ejection fraction; *LAVI* left atrial volumetric index; *LVEF* left ventricular ejection fraction; LVMI left ventricular mass index; *MRA* mineralocorticoid receptor antagonist; *NT-proBNP* N-terminal pro B‑type natriuretic peptide; *NYHA* New York Heart Association; *RVD* right ventricular dysfunction; *SBP* systolic blood pressure

§* p0.05* versus HFrEF; †* p0.05 *versus HFmrEF

### Baseline characteristics by BMI, EAT, and PAT

The mean BMI in the entire cohort was 27 ± 6 kg/m^2^, mean EAT thickness was 4.8 ± 2.1 mm and the mean PAT thickness was 5.7 ± 2.8 mm. As demonstrated in the Electronic Supplemental Material (ESM) Figure S1, EAT and PAT thickness ranged from 1.3 to 22.8 mm and 1.3 to 20.8 mm, respectively.

Tab. 1 and Figure S2 showed that HFpEF patients had significantly higher EAT and PAT thickness than patients with HFrEF and HFmrEF.

Tables S1 and *S*2 showed that patients with higher EAT and PAT thickness had significantly higher BMI and were more likely to be older. In addition, patients with both higher EAT and PAT thickness had ischaemic heart disease significantly more often, while LVEF was higher. These patients also had a lower glomerular filtration rate (eGFR) (Tables S1 and S2). Finally, Figure S3 shows that women had significantly higher EAT thickness, whereas men had higher PAT thickness.

In contrast, patients with high BMI did not show a significantly higher prevalence of ischaemic heart disease or lower eGFR (Table S3). Conversely, patients with a high BMI were more often women and more frequently had hypertension, diabetes mellitus, and atrial fibrillation, but also had higher LVEF (Table S3).

### Echocardiographic measures

Table S1 and S2 showed that most echocardiographic parameters, such as E/e′ and ventricular mass index, did not differ across EAT and PAT quartiles. However, higher PAT thickness was significantly associated with lower left atrial volume and lower lateral e′ (*p=0.019* and *p=0.022*, respectively). Patients with high BMI had lower left atrial volume index and higher septal and lateral e′ (Table S3).

### Clinical outcomes by BMI, EAT, and PAT

**Fig. 3:**
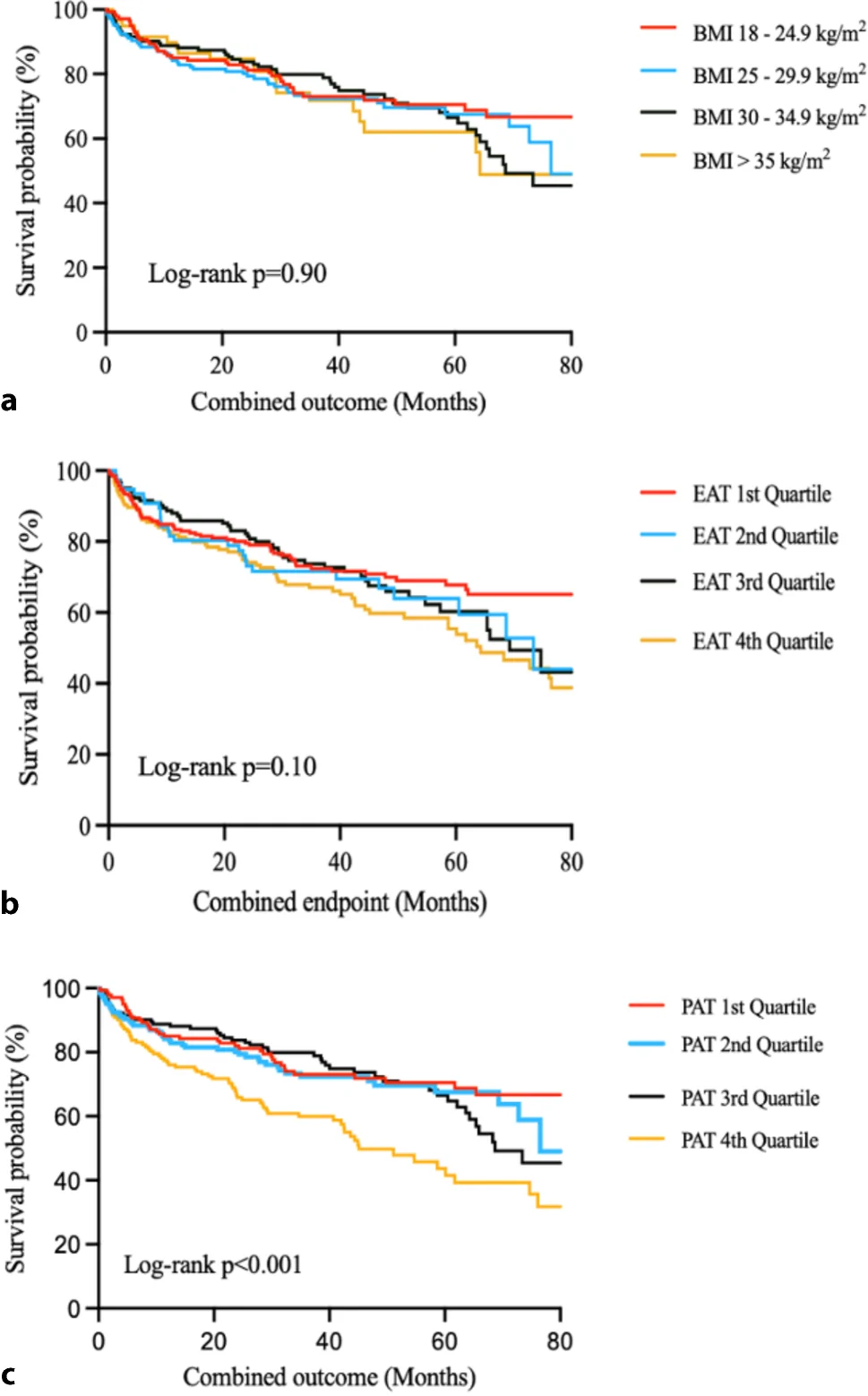
Associations between body mass index, epicardial and pericardial adipose tissue with outcome. Kaplan Meier curves depicted for the survival of combined outcome. BMI groups (**a**) *p* = 0.9; EAT quartiles (**b**) *p* = 0.04; PAT quartiles (**c**) *p*  0.001. *BMI* body mass index; *COPD* chronic obstructive pulmonary disease; *EAT* epicardial adipose tissue; *HF* heart failure; *HFrEF* heart failure with reduced ejection fraction; *HFmrEF* heart failure with mildly reduced ejection fraction; *HFpEF* heart failure with preserved ejection fraction; *PAT* pericardial adipose tissue

**Fig. 4:**
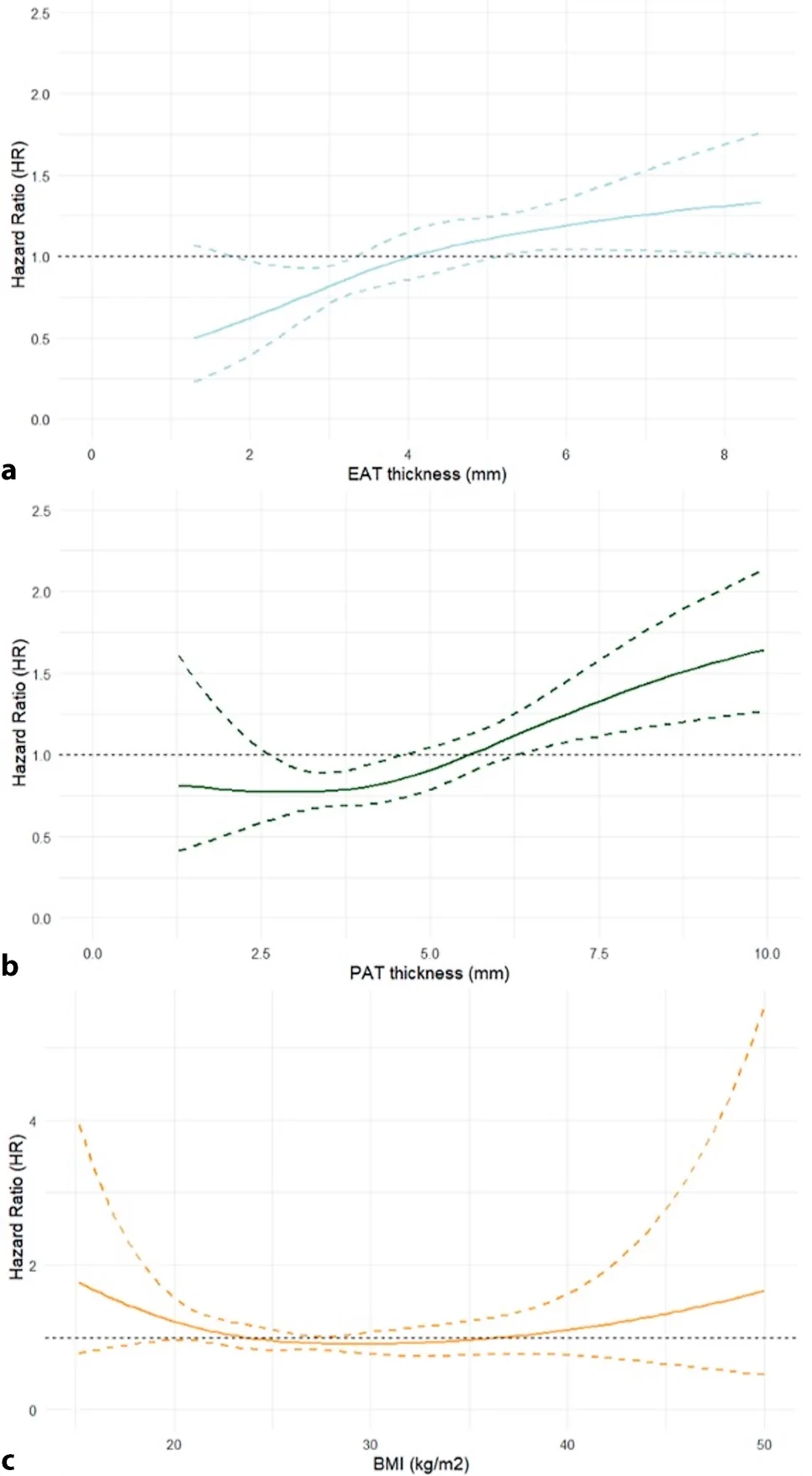
Spline curves for the hazard ratio of the combined outcome. This figure shows the relationship between the hazard ratio of the combined outcome and **a** EAT, **b** PAT and **c** BMI. Panels A and B were displayed up to the 95th percentile of the distribution. Odds ratio (*solid line*) and 95% confidence intervals (*colored dashed lines*) were based on the Cox proportinal hazard model. *BMI* body mass index; *EAT* epicardial adipose tissue; *PAT* pericardial adipose tissue

In total, 217 patients (35%) experienced the combined outcome during a mean follow-up of 3.2 ± 1.9 years. Univariate Cox regression analysis demonstrated that higher EAT and PAT thickness were associated with a higher risk of the combined outcome [HR 1.15 (95% CI 1.03–1.30), *p=* 0.013; HR 1.25 (95% CI 1.10–1.41), *p*  0.001, respectively]; Tab. 2. Furthermore, for both EAT and PAT, this association remained significant after adjustment for BMI. However, the only association with PAT remained significant in the multivariate regression model 2, when adjusted for age, sex, BMI, HF type, and 14 additional univariably predictors of outcome [HR 1.49 (95% CI 1.10–2.03), *p=* 0.008], whereas this association was not seen for EAT thickness (Tab. 2). Moreover, restricted spline analyses showed approximately linear associations between EAT and PAT thickness with the risk of the combined outcome, with increasing EAT and PAT thickness associated with a higher HR (Fig. 4a and 3b). The prognostic cutoff for PAT was approximately  6 mm and for EAT approximately  5 mm. In contrast, a U-shaped association was observed between BMI and the combined outcome, as shown in Fig. 4c.

**Table 2:** Univariate and multivariate Cox regression modelling.

			Combined outcome			
	Univariate HR (95% CI)	*p*-value	Multivariate HR (95% CI) Model 1	*p*-value	Multivariate HR (95% CI) Model 2	*p*-value
Age, years	1.06 (1.04–1.07)	**< 0.001**	1.06 (1.05–1.07)	**< 0.001**	1.02 (0.98–1.05)	0.1
Sex, Women	1.16 (0.89–1.52)	0.3				
Body mass index (kg/m^2^)	1.00 (0.97–1.02)	0.8				
*HF characteristics*						
HF type	1.19 (1.02–1.39)	** 0.028**	1.20 (1.03–1.41)	** 0.019**	1.26 (0.87–1.84)	0.2
Ischaemic heart disease	1.49 (1.14–1.94)	** 0.003**	1.50 (1.14–1.94)	** 0.003**	1.67 (0.92–3.03)	0.094
NYHA Class	3.10 (2.37–4.06)	**< 0.001**			1.48 (0.92–2.34)	0.1
*Comorbidities*						
Hypertension	1.32 (1.01–1.73)	** 0.044**	1.38 (1.08–1.82)	** 0.025**	1.21 (0.68–2.16)	0.5
Atrial fibrillation, history	1.37 (1.05–1.78)	** 0.022**	1.38 (1.05–1.82)	** 0.020**	1.0 (0.5–2.1)	1.0
Diabetes mellitus	1.60 (1.19–2.14)	** 0.002**	1.71 (1.26–2.30)	**< 0.001**	1.89 (1.01–3.50)	** 0.048**
COPD	2.03 (1.47–2.78)	**< 0.001**	1.99 (1.44–2.78)	**< 0.001**	2.89 (1.43–5.71)	** 0.003**
Cardiothoracic surgery	1.54 (1.10–2.15)	** 0.011**	1.51 (1.08–2.10)	** 0.016**	1.03 (0.50–2.12)	0.9
*Laboratory tests*						
eGFR, ml/min/1.73 m2	0.41 (0.34–0.51)	**< 0.001**	0.45 (0.37–0.56)	**< 0.001**	0.50 (0.22–1.16)	0.2
NT-proBNP, ng/l	1.67 (1.48–1.89)	**< 0.001**	1.72 (1.52–1.94)	**< 0.001**	1.72 (1.50–2.00)	**< 0.001**
*Medication*						
Beta-blocker use	1.13 (0.89–1.96)	0.2				
ACEi/ARB use	0.78 (0.56–1.09)	0.2				
Loop diuretic use	2.16 (2.16–3.00)	**< 0.001**	2.80 (1.92–4.19)	**< 0.001**	1.06 (0.51–2.20)	0.9
*Echocardiographic parameters*						
LVEF (%)	1.01 (1.00–1.02)	0.068				
LAVI	1.02 (1.01–1.02)	** 0.002**	1.02 (1.01–1.02)	** 0.003**	1.02 (1.00–1.04)	** 0.034**
LVMI	1.01 (1.00–1.01)	0.6				
e′ lateral	0.96 (0.91–1.01)	0.1				
e′ septal	0.87 (0.79–0.94)	**< 0.001**	0.82 (0.67–0.95)	**< 0.001**	0.78 (0.61–1.10)	0.064
e′ mean	0.92 (0.86–0.99)	** 0.027**	0.92 (0.86–0.99)	** 0.025**	1.22 (0.95–1.57)	0.1
E/e′	1.09 (1.05–1.12)	**< 0.001**	1.09 (1.05–1.12)	**< 0.001**	1.04 (0.98–1.12)	0.2
EAT thickness, mm	1.15 (1.03–1.30)	** 0.013**	1.18 (1.05–1.32)	** 0.006**	1.07 (0.87–1.32)	0.5
PAT thickness, mm	1.25 (1.10–1.41)	**< 0.001**	1.27 (1.13–1.44)	**< 0.001**	1.49 (1.10–2.03)	** 0.008**

*CI* Confidence interval (Logarithmic scale for NT-pro-BNP and eGFR; Per SD for EAT and PAT) *EAT* epicardial adipose tissue; *HR* hazard ratio; *PAT* pericardial adipose tissue

Multivariate model 1 is used to adjust for BMI. Multivariate model 2 is used to adjust for age, sex, BMI, HF type and all univariate associated variables (i.e. ischaemic heart disease, NYHA class, eGFR, NT-proBNP, loop diuretic use, LAVI, e′ septal, e′ mean, E/e′ and presence/history of hypertension, atrial fibrillation, diabetes mellitus, COPD and cardiothoracic surgery)

In Fig. 3a, Kaplan-Meier curves survival-free period of the combined outcome are depicted. No association with the combined outcome was observed across the BMI groups or EAT quartiles (Log-rank *p* = 0.90; Fig. 3a and Log-rank *p* = 0.10; Fig. 3b, respectively). Conversely, patients with higher PAT thickness were significantly at risk for the combined outcome (Log-rank *p*  0.001, respectively; Fig. 3c). Furthermore, Figure S4 demonstrated Kaplan-Meier curves for the combined outcome, stratified by the HF groups. Patients with high PAT thickness within the HFrEF group and especially the HFpEF subgroup were significantly more at risk for the combined outcome than patients with lower PAT thickness (Log-rank *p* = 0.019 *and p* = 0.008, respectively), whereas this association was not present in patients with HFmrEF. In addition, no differences were observed according to EAT quartiles within the HF groups (Figure S5).

## Discussion

The main finding of the present study was that both EAT and PAT thickness, measured by standard echocardiography, were associated with poor outcomes in patients with established HF, whereas this association was not observed for higher BMI. We also demonstrated that these AT depots were differentially associated with outcomes across HF phenotypes, including HFrEF, HFmrEF, and HFpEF. Additionally, we showed that patients with high EAT and PAT thickness had different clinical characteristics compared to patients with high BMI. These findings suggest that increased EAT and PAT thickness are independently associated with poor clinical outcomes in patients with HF and predominantly in HFpEF.

The association observed between high EAT thickness and an increased risk of all-cause mortality and HF hospitalization aligns with previous studies [16, 17]. For instance, Pugliese et al. demonstrated in a cohort of 188 HFpEF patients that high EAT thickness was significantly associated with an increased risk for HF hospitalization and cardiovascular death [16]. However, we have now demonstrated similar findings in a larger, all-comer HF population. It is suggested that EAT, in physiological conditions, can serve as a metabolic reservoir due to its protective brown-fat features. However, excessive EAT accumulation may induce a shift towards a pro-inflammatory phenotype characterized by the release of a broad range of adipocytokines that may damage the myocardium and subsequently cause, e.g., HF and poor outcomes [18, 19]. Additionally, it was seen that EAT thickness was significantly higher in HFpEF patients than their HFrEF counterparts, which was in line with a previous study [20].

To date, less attention has been given in clinical research to the potential associations between PAT and clinical outcome [21]. However, interest is increasing, as colleagues from the SUMMIT trial have recently demonstrated that Tirzepatide significantly reduces PAT, more than EAT [22]. In the present study, we have demonstrated that higher PAT thickness was significantly associated with an increased risk of the combined outcome independent of BMI, age, sex, and other variables associated with outcome in univariable analysis. It has been hypothesized that an increased burden of PAT promotes multiple deleterious metabolic processes, including insulin resistance, aldosterone secretion, and systemic inflammation [22, 23]. Ultimately, this may result in a higher risk of poor outcome, whereas PAT thickness can thus be used as an important and easily obtainable prognostic marker in chronic HF [24].

### Clinical implications

Recent studies have advocated the use of alternative anthropometric measures such as waist-to-hip ratio, waist-to-height ratio, and body roundness index [4, 25]. Sub-studies from the PARAGON-HF and PARADIGM-HF trials demonstrated that these anthropometric measures more closely reflect the amount and distribution of body fat and provide prognostic value in HF, beyond overall obesity [4, 23, 25, 26]. However, these anthropometric measures do not account for the characteristics of different fat depots and only provide an estimate of ‘central obesity’ [6]. Unlike these anthropometric measures, EAT and PAT reflect local fat accumulation and may directly influence the myocardium due to their metabolically active and pro-inflammatory profile. Therefore, they may provide a more accurate reflection of cardiovascular risk and yield additional clinical relevance.

Although CMR is more precise for the quantification of EAT and PAT volumes, standard echocardiography may be a useful bedside alternative to easily assess the thickness of these fat depots [27]. Although echocardiography cannot measure the entire volume of EAT and PAT, the assessment of both EAT and PAT based on a thickness on 2D echocardiography should therefore be regarded as an easily obtainable, anthropometric measurement that may serve as an important screening tool in patients with HF [16, 28]. For PAT thickness, the prognostic cutoff point was approximately  6 mm, and for EAT this was  5 mm.

### Limitations

This study has several limitations. First, it was conducted in a tertiary HF referral centre, which may have introduced referral bias, and most of the study population included HFrEF patients. Second, we did not have data to adjust for visceral adipose tissue, and anthropometric measures such as waist-to-hip ratio and waist-to-height ratio were also not available. Third, CMR and computed tomography (CT) generally provide a more precise quantification of the total amount of EAT and PAT. However, CMR and CT are often not readily available in clinical practice. In contrast, routine echocardiography provides a quick and easily accessible alternative for assessing AT thickness, which may serve as an important prognostic marker, although it is limited by lower resolution and inability to capture total fat volume [27, 29].

## Conclusion

This study demonstrates that both EAT and PAT thickness were associated with poor outcomes, irrespective of BMI, in an unselected population of HF. In a multivariable model, only PAT thickness remained significantly associated with poor outcome. The association between PAT thickness and poor outcome was mainly observed in HFpEF. These data suggest that PAT thickness, more than EAT, may serve as an easily obtainable prognostic marker in patients with HF, especially HFpEF and irrespective of overall obesity.

## Supplementary Information

ESM1: Supplementary material 1

## Data Availability

All data supporting the findings of this work are included in the article.

## References

[1] Rubino F, Cummings DE, Mingrone G, et al. Definition and diagnostic criteria of clinical obesity. Lancet Diabetes Endocrinol. 2025;13:221–62. 10.1016/S2213-8587(24)00316-4 PMC11870235 39824205

[2] Kenchaiah S, Evans JC, Vasan RS, et al. Obesity and the risk of heart failure. N Engl J Med. 2002;347:305–13. 10.1056/NEJMoa020245 12151467

[3] Aune D, Sen A, Vatten LJ, et al. Body Mass Index, Abdominal Fatness, and Heart Failure Incidence and Mortality: A Systematic Review and Dose-Response Meta-Analysis of Prospective Studies. Circulation. 2016;133:639–49. 10.1161/CIRCULATIONAHA.115.016801 26746176

[4] Butt JH, Petrie MC, McMurray JJV, et al. Anthropometric measures and adverse outcomes in heart failure with reduced ejection fraction: revisiting the obesity paradox. Eur Heart J. 2023;44:1136–53. 10.1093/eurheartj/ehad083 PMC10111968 36944496

[5] Streng KW, Voors AA, Lang CC, et al. Waist-to-hip ratio and mortality in heart failure. Eur J Heart Fail. 2018;20:1269–77. 10.1002/ejhf.1244 29963737

[6] Lobeek M, Timmann LPA, Gorter TM, et al. Epicardial, visceral and subcutaneous adipose tissue in heart failure with preserved ejection fraction. ESC Heart Failure. 2025;12:3353–60. 10.1002/ehf2.15396 PMC12450799 40827329

[7] Wong CX, Mahajan R, Pathak R, et al. The Role of Pericardial and Epicardial Fat in Atrial Fibrillation Pathophysiology and Ablation Outcomes. J Atr Fibrillation. 2013;5:790. 10.4022/jafib.790 PMC5153111 28496816

[8] Iacobellis G. Local and systemic effects of the multifaceted epicardial adipose tissue depot. Nat Rev Endocrinol. 2015;11:363–71. 10.1038/nrendo.2015.58 25850659

[9] Crum Y, van Veldhuisen DJ, Gorter TM, et al. Epicardial adipose tissue, functional status and invasive hemodynamics in heart failure with preserved ejection fraction. JACC Adv. 2025:102185. 10.1016/j.jacadv.2025.102185 PMC12793855 40892622

[10] Dronkers J, van Veldhuisen DJ, van der Meer P, et al. Heart Failure and Obesity: Unraveling Molecular Mechanisms of Excess Adipose Tissue. J Am Coll Cardiol. 2024;84:1666–77. 10.1016/j.jacc.2024.07.016 39415402

[11] Nauta JF, Santema BT, Voors AA, et al. Improvement in left ventricular ejection fraction after pharmacological up-titration in new-onset heart failure with reduced ejection fraction. Neth Heart J. 2021;29:383–93. 10.1007/s12471-021-01591-6 PMC8271074 34125353

[12] Ansari Ramandi MM, van Melle JP, Dickinson MG, et al. Right ventricular dysfunction in patients with new-onset heart failure: longitudinal follow-up during guideline-directed medical therapy. Eur J Heart Fail. 2022;24:2226–34. 10.1002/ejhf.2721 PMC10099924 36250250

[13] Ponikowski P, Voors AA, van der Meer P, et al. 2016 ESC Guidelines for the diagnosis and treatment of acute and chronic heart failure. Kardiol Pol. 2016;74:1037–147. 10.5603/KP.2016.0141 27748494

[14] Iacobellis G. Epicardial adipose tissue in contemporary cardiology. Nat Rev Cardiol. 2022;19:593–606. 10.1038/s41569-022-00679-9 PMC8926097 35296869

[15] Schmidt M, Ulrichsen SP, Pedersen L, Bøtker HE, Sørensen HT. Thirty-year trends in heart failure hospitalization and mortality rates and the prognostic impact of co-morbidity: a Danish nationwide cohort study. Eur J Heart Fail. 2016;18:490–9. 10.1002/ejhf.486 26868921

[16] Pugliese NR, Paneni F, Masi S, et al. Impact of epicardial adipose tissue on cardiovascular haemodynamics, metabolic profile, and prognosis in heart failure. Eur J Heart Fail. 2021;23:1858–71. 10.1002/ejhf.2337 34427016

[17] van Woerden G, van Veldhuisen DJ, Gorter TM, et al. Epicardial Adipose Tissue and Outcome in Heart Failure With Mid-Range and Preserved Ejection Fraction. Circ Heart Fail. 2022;15:e009238. 10.1161/CIRCHEARTFAILURE.121.009238 PMC8920003 34935412

[18] Cherian S, Lopaschuk GD, Carvalho E. Cellular cross-talk between epicardial adipose tissue and myocardium in relation to the pathogenesis of cardiovascular disease. Am J Physiol Endocrinol Metab. 2012;303:E937–E49. 10.1152/ajpendo.00061.2012 22895783

[19] Iozzo P. Myocardial, perivascular, and epicardial fat. Diabetes Care. 2011;34(Suppl 2):S371–S9. 10.2337/dc11-s250 PMC3632210 21525485

[20] Jin X, Hung CL, Lam CSP, et al. Epicardial adipose tissue related to left atrial and ventricular function in heart failure with preserved versus reduced and mildly reduced ejection fraction. Eur J Heart Fail. 2022;24:1346–56. 10.1002/ejhf.2513 35475591

[21] Ahmed B, Sultana R, Greene MW. Adipose tissue and insulin resistance in obese. Biomed Pharmacother. 2021;137:111315. 10.1016/j.biopha.2021.111315 33561645

[22] Kramer C, Borlaug B, Zile M, et al. Tirzepatide Reduces LV Mass and Paracardiac Adipose Tissue in Obesity-Related Heart Failure: SUMMIT CMR Substudy. JACC. 2025;85:699–706. 10.1016/j.jacc.2024.11.001 39566869

[23] Peikert A, Vaduganathan M, Packer M, et al. Near-universal prevalence of central adiposity in heart failure with preserved ejection fraction: the PARAGON-HF trial. Eur Heart J. 2025;46:2372–90. 10.1093/eurheartj/ehaf057 PMC12208775 39873282

[24] Ardissino M, McCracken C, Raisi-Estabragh Z, et al. Pericardial adiposity is independently linked to adverse cardiovascular phenotypes: a CMR study of 42 598 UK Biobank participants. Eur Heart J. Cardiovasc Imaging. 2022;23:1471–81. 10.1093/ehjci/jeac101 PMC9584621 35640889

[25] Clark AL, Fonarow GC, Horwich TB. Waist circumference, body mass index, and survival in systolic heart failure: the obesity paradox revisited. J Card Fail. 2011;17:374–80. 10.1016/j.cardfail.2011.01.009 21549293

[26] Loehr LR, Rosamond WD, Heiss G, et al. Association of multiple anthropometrics of overweight and obesity with incident heart failure: the Atherosclerosis Risk in Communities study. Circ Heart Fail. 2009;2:18–24. 10.1161/CIRCHEARTFAILURE.108.813782 PMC2748859 19808311

[27] van Woerden G, van Veldhuisen DJ, Westenbrink BD, et al. The value of echocardiographic measurement of epicardial adipose tissue in heart failure patients. ESC Heart Fail. 2022;9:953–7. 10.1002/ehf2.13828 PMC8934911 35146949

[28] Chen LQ, Scheiner J, Cao JJ, et al. Epicardial fat modifies the relationship between coronary calcium score and all-cause mortality: The St. Francis Heart Study. Am J Prev Cardiol. 2024;19:100689. 10.1016/j.ajpc.2024.100689 PMC11245900 39005754

[29] Guglielmo M, Lin A, Pontone G, et al. Epicardial fat and coronary artery disease: Role of cardiac imaging. Atherosclerosis. 2021;321:30–8. 10.1016/j.atherosclerosis.2021.02.008 33636676

