# Supplementary material for: Prognostic value of epicardial and pericardial adipose tissue in patients with chronic heart failure
Source: Neth Heart J. 2026 Sep 16;34(10):369–79. doi: 10.1007/s12471-026-02077-z (PMC13615238; doi:10.1007/s12471-026-02077-z)
Supplement: Supplementary file 1 [file 12471_2026_2077_MOESM1_ESM.docx]

**SUPPLEMENTARY MATERIAL**

**Prognostic value of epicardial and pericardial adipose tissue in patients with chronic heart failure**

*Y. Crum et al.*

**Supplemental Figure 1: Distribution of EAT and PAT**

This figure shows the frequency distribution curves of EAT (A) and PAT (B) thickness according to sex. The orange line and bars represent women and the blue line and bars represent men. EAT, epicardial adipose tissue; PAT, pericardial adipose tissue

**Supplemental Figure 2: Association of EAT and PAT within different groups of heart failure** Figure A reflects the association with EAT and HF groups; Figure B reflects the association between PAT and HF groups. Error bars reflect the standard error of the mean. *EAT, epicardial adipose tissue;HFrEF, heart failure with reduced ejection fraction; HFmrEF, heart failure with mildly reduced ejection fraction; HFpEF, heart failure with preserved ejection fraction; PAT, pericardial adipose tissue*

Q

**Supplemental Figure 3: Difference in EAT and PAT between men and women** Error bars reflect standard error of the mean *EAT, epicardial adipose tissue; PAT, pericardial adipose tissue*

**Supplemental Figure 4: Kaplan-Meier curves for the combined outcome** Survival status PAT quartiles in HF groups. HFrEF (A), HFmrEF (B), HFpEF (C). *HFpEF, heart failure with preserved ejection fraction; HFmrEF, heart failure with mildy reduced ejection fraction; HFrEF, heart failure with reduced ejection fraction; PAT, pericardial adipose tissue*

**Supplemental Figure 5: Kaplan-Meier curves for the combined outcome** Survival status EAT quartiles in HF groups. HFrEF (A), HFmrEF (B), HFpEF (C). *EAT, epicardial adipose tissue; HFpEF, heart failure with preserved ejection fraction; HFmrEF, heart failure with mildy reduced ejection fraction; HFrEF, heart failure with reduced ejection fraction;*

**Supplementary Table 1: Baseline table EAT; demographics, physical examination, comorbidities, laboratory test and echocardiographic parameters**

|  | **Total**  **(n=572)** | **EAT 1^st^ Quartile (n=145)** | **EAT 2^nd^ Quartile (n=140)** | **EAT 3^rd^ Quartile (n=142)** | **EAT 4^th^ Quartile**  **(n=145)** | **P for trend** |
| --- | --- | --- | --- | --- | --- | --- |
| Age, years | 67 ± 14 | 63 ± 17 | 67 ± 13 | 68 ± 13 *§* | 72 ± 11 *§†* | **<0.001** |
| Sex, females % | 217 (38%) | 51 (35%) | 52 (36%) | 51 (35%) | 71 (50%) *§†‡* | 0.032 |
| Body mass index (kg/m^2^) | 27 ± 6 | 25 ± 5 | 27 ± 5 *§* | 28 ± 5 *§* | 30 ± 6 *§†‡* | **<0.001** |
| *HF characteristics* |  |  |  |  |  |  |
| HF type   - HFrEF - HFmrEF - HFpEF | 346 (60%)  100 (17%)  126 (22%) | 109 (75%)  14 (10%)  22 (15%) | 94 (66%)  29 (20%)  20 (14%) | 79 (55%) *§*  32 (22%)  32 (22%) | 70 (49%) *§†*  21 (15%)  52 (36%) | **<0.001** |
| Ischaemic heart disease | 237 (38%) | 42 (29%) | 48 (34%) | 54 (38%) | 72 (50%) *§†‡* | **0.001** |
| NYHA Class   - I - II - III - IV | 182 (30%)  325 (52%)  100 (16%)  7 (1%) | 28 (20%)  72 (50%)  41 (28%)  2 (1%) | 24 (17%)  76 (53%)  40 (28%)  2 (1%) | 19 (13%)  80 (56%)  37 (26%)  2 (1%) | 12 (8%) *§†*  73 (51%)  55 (38%)  2 (1%) | 0.2 |
| *Comorbidities* |  |  |  |  |  |  |
| Hypertension | 216 (34%) | 40 (28%) | 50 (35%) | 49 (34%) | 60 (42%) | 0.086 |
| Atrial fibrillation, history | 241 (38%) | 50 (35%) | 51 (37%) | 58 (41%) | 54 (38%) | 0.7 |
| Diabetes mellitus | 138 (22%) | 26 (18%) | 32 (22%) | 26 (18%) | 40 (28%) | 0.1 |
| COPD | 85 (14%) | 9 (6%) | 14 (10%) | 18 (13%) *§* | 32 (22%) *§†* | **<0.001** |
| Cardiothoracic surgery | 94 (15%) | 20 (14%) | 25 (18%) | 22 (15%) | 18 (13%) | 0.676 |
| *Laboratory tests* |  |  |  |  |  |  |
| eGFR, ml/min/1.73 m2 | 67 ± 25 | 72 ± 25 | 67 ± 27 | 66 ± 24 | 59 ± 23 *§* | **<0.001** |
| NT‐proBNP, ng/l | 1538 [558-3679] | 1526 [525 – 3869] | 1351 [538 – 3084] | 1461 [542 – 2688] | 1461 [542 – 2688] | 0.7 |
| *Medication* |  |  |  |  |  |  |
| Beta‐blocker use | 534 (85%) | 120 (83%) | 120 (84%) | 125 (87%) | 128 (90%) | 0.3 |
| ACEi/ARB use | 521 (83%) | 126 (87%) | 123 (86%) | 120 (84%) | 115 (80%) | 0.4 |
| MRA use | 242 (39%) | 86 (47%) | 58 (41%) | 57 (40%) | 41 (29%) *§* | **0.015** |
| Loop diuretic use | 437 (70%) | 96 (66%) | 96 (67%) | 94 (66%) | 112 (78%) | 0.063 |
| *Echocardiographic parameters* |  |  |  |  |  |  |
| LVEF (%) | 38 ± 13 | 34 ± 12 | 34 ± 13 | 37 ± 14 | 42 ± 13 *§†‡* | **<0.001** |
| LAVI | 43 ± 15 | 46 ± 15 | 40 ± 15 | 44 ± 13 | 41 ± 16 | 0.2 |
| LVMI | 107 ± 32 | 107 ± 36 | 105 ± 39 | 109 ± 30 | 109 ± 25 | 0.9 |
| e’ lateral | 8.3 ± 3.2 | 8.6 ± 3.6 | 8.3 ± 3.5 | 8.0 ± 3.1 | 8.1 ± 2.8 | 0.2 |
| e’ septal | 5.9 ± 2.3 | 6.0 ± 2.5 | 6.1 ± 2.3 | 5.6 ± 2.1 | 6.0 ± 2.2 | 0.2 |
| e’ mean | 7.1 ± 2.5 | 7.3 ± 2.7 | 7.2 ± 2.7 | 6.9 ± 2.3 | 7.1 ± 2.3 | 0.3 |
| E/e’ | 12 ± 5 | 11 ± 6 | 12 ± 6 | 12 ± 5 | 12 ± 5 | 0.5 |
| EAT | 4.8 ± 2.1 | 3.0 ± 0.6 | 4.1 ± 0.2 *§* | 5.0 ± 0.5 *§†* | 7.5 ± 1.9 *§†‡* | **<0.001** |
| PAT | 5.7 ± 2.8 | 4.0 ± 1.8 | 5.8 ± 2.6 *§* | 6.1 ± 2.7 *§* | 7.3 ± 3.1 *§†‡* | **<0.001** |

Data are mean±SD, median [interquartile range] or n (%). Final column reflects overall differences in EAT tertiles. ANOVA was used for normally distributed parameters and Chi-squared was used for categorical parameters*.
ACEi, angiotensin‐converting enzyme inhibitor; ARB, angiotensin receptor blocker; BMI, body mass index; COPD, chronic obstructive pulmonary disease; DBP, diastolic blood pressure; eGFR, estimated glomerular filtration rate; HF, heart failure; HFrEF, heart failure with reduced ejection fraction; HFmrEF, heart failure with mildly reduced ejection fraction; HFpEF, heart failure with preserved ejection fraction; LAVI, left atrial volumetric index; LVEF, left ventricular ejection fraction; LVMI, left ventricular mass index; MRA, mineralocorticoid receptor antagonist; NT‐proBNP, N‐terminal pro B‐type natriuretic peptide; NYHA, New York Heart Association; RVD, right ventricular dysfunction; SBP, systolic blood pressure § p<.05 versus 1st quartile; † p<.05 versus 2nd quartile; ‡ p<.05 verus 3rd quartile*

**Supplementary Table 2: Baseline table PAT; demographics, physical examination, comorbidities, laboratory test and echocardiographic parameters**

|  | **Total**  **(n=572)** | **PAT 1^st^ quartile (n=140)** | **PAT 2^nd^ quartile**  **(n=146)** | **PAT 3^rd^ quartile (n=143)** | **PAT 4^th^ quartile**  **(n=143)** | **P for trend** |
| --- | --- | --- | --- | --- | --- | --- |
| Age, years | 67 ± 14 | 60 ± 18 | 67 ±12 *§* | 68 ± 14 *§* | 72 ± 10 *§* | **<0.001** |
| Sex, females % | 217 (38%) | 67 (48%) | 55 (38%) | 48 (34%) *§* | 54 (38%) | 0.087 |
| Body mass index (kg/m^2^) | 27 ± 6 | 25 ± 5 | 27 ± 5 *§* | 28 ± 6 *§* | 29 ± 6 *§†* | **<0.001** |
| *HF characteristics* |  |  |  |  |  |  |
| HF type   - HFrEF - HFmrEF - HFpEF | 346 (60%)  100 (17%)  126 (22%) | 87 (62%)  24 (17%)  29 (21%) | 94 (64%)  27 (19%)  25 (17%) | 96 (67%)  23 (16%)  24 (17%) | 73 (51%) *‡*  22 (15%)  48 (34%) | **0.013** |
| Ischaemic heart disease | 237 (38%) | 35 (25%) | 61 (42%) *§* | 63 (44%) *§* | 57 (40%) *§* | **0.004** |
| NYHA Class  I  II  III  IV | 182 (30%)  325 (52%)  100 (16%)  7 (1%) | 30 (22%)  61 (43%)  46 (1633  1 (1%) | 20 (14%)  87 (60%)  33 (23%)  4 (3%) | 19 (13%)  83 (58%)  37 (26%)  2 (1%) | 14 (10%) *§*  69 (48%)  56 (39%)  2 (1%) | **0.010** |
| *Comorbidities* |  |  |  |  |  |  |
| Hypertension | 216 (34%) | 38 (27%) | 49 (34%) | 54 (38%) | 57 (40%) | 0.1 |
| Atrial fibrillation, history | 241 (38%) | 50 (36%) | 59 (40%) | 41 (29%) | 63 (44%) *§‡* | **0.044** |
| Diabetes mellitus | 138 (22%) | 22 (16%) | 38 (26%) | 26 (18%) | 37 (26%) | 0.069 |
| COPD | 85 (14%) | 10 (7%) | 19 (13%) | 14 (10%) | 29 (20%) *§‡* | **0.006** |
| Cardiothoracic surgery | 94 (15%) | 19 (14%) | 24 (16%) | 25 (18%) | 17 (12%) | 0.523 |
| *Laboratory tests* |  |  |  |  |  |  |
| eGFR, ml/min/1.73 m2 | 67 ± 25 | 73 ± 27 | 68 ± 25 | 65 ± 25 *§* | 60 ± 23 *§* | **<0.001** |
| NT‐proBNP, ng/l | 1538 [558-3679] | 1538 [558-3679] | 1349 [452-3022] | 1396 [590-2953] | 1310 [562 – 2577] | 0.9 |
| *Medication* |  |  |  |  |  |  |
| Beta‐blocker use | 534 (85%) | 112 (80%) | 128 (88%) | 126 (88%) | 125 (87%) | 0.2 |
| ACEi/ARB use | 521 (83%) | 124 (89%) | 128 (89%) | 118 (83%) | 112 (79%) | 0.061 |
| MRA use | 242 (39%) | 61 (44%) | 62 (43%) | 48 (34%) | 51 (36%) | 0.2 |
| Loop diuretic use | 437 (70%) | 86 (61%) | 108 (74%) | 98 (69%) | 104 (73%) | 0.094 |
| *Echocardiographic parameters* |  |  |  |  |  |  |
| LVEF (%) | 38 ± 13 | 37 ± 12 | 35 ± 13 | 35 ± 13 | 40 ± 14 *†‡* | **0.010** |
| LAVI | 43 ± 15 | 47 ± 18 | 42 ± 13 | 43 ± 16 | 41 ± 12 *§* | **0.019** |
| LVMI | 107 ± 32 | 105 ± 36 | 110 ± 32 | 108 ± 34 | 107 ± 27 | 0.7 |
| e’ lateral | 8.3 ± 3.2 | 9.1 ± 3.8 | 8.1 ± 3.4 | 7.7 ± 2.9 *§* | 8.1 ± 3.1 | **0.022** |
| e’ septal | 5.9 ± 2.3 | 6.3 ± 2.7 | 5.6 ± 2.2 | 5.6 ± 1.8 | 6.1 ± 2.3 | 0.066 |
| e’ mean | 7.1 ± 2.5 | 7.7 ± 2.9 | 7.0 ± 2.6 | 6.8 ± 2.0 | 7.1 ± 2.5 | 0.054 |
| E/e’ | 12 ± 5 | 11 ± 6 | 12 ± 6 | 11 ± 5 | 12 ± 5 | 0.5 |
| EAT | 4.8 ± 2.1 | 3.5 ± 1.8 | 4.5 ± 1.5 *§* | 5.0 ± 1.5 *§* | 6.1 ± 2.5 *§†‡* | **<0.001** |
| PAT | 5.7 ± 2.8 | 2.8 ± 0.7 | 4.3 ± 0.4 *§* | 5.8 ± 0.5 *§†* | 9.4 ± 2.6 *§†‡* | **<0.001** |

Data are mean±SD, median [interquartile range] or n (%). Final column reflects overall differences in EAT tertiles. ANOVA was used for normally distributed parameters and Chi-squared was used for categorical parameters*.
ACEi, angiotensin‐converting enzyme inhibitor; ARB, angiotensin receptor blocker; BMI, body mass index; COPD, chronic obstructive pulmonary disease; DBP, diastolic blood pressure; eGFR, estimated glomerular filtration rate; HF, heart failure; HFrEF, heart failure with reduced ejection fraction; HFmrEF, heart failure with mildly reduced ejection fraction; HFpEF, heart failure with preserved ejection fraction; LAVI, left atrial volumetric index; LVEF, left ventricular ejection fraction; LVMI, left ventricular mass index; MRA, mineralocorticoid receptor antagonist; NT‐proBNP, N‐terminal pro B‐type natriuretic peptide; NYHA, New York Heart Association; § p<.05 versus 1st quartile; † p<.05 versus 2nd quartile; ‡ p<.05 verus 3rd quartile*

**Supplementary Table 3: Baseline table BMI; demographics, physical examination,**

**comorbidities, laboratory test and echocardiographic parameters**

|  | **Total**  **(n=572)** | **BMI**  **<25 kg/m^2^**  **(n=233)** | **BMI**  **25-30 kg/m^2^**  **(n=214)** | **BMI**  **30-35 kg/m^2^**  **(n=111)** | **BMI**  **>35 kg/m^2^**  **(n=58)** | **P for trend** |
| --- | --- | --- | --- | --- | --- | --- |
| Age, years | 67 ± 14 | 67 ± 16 | 67 ± 14 | 67 ± 13 | 67 ± 13 | 1.0 |
| Sex, females % | 217 (38%) | 101 (43%) | 61 (29%) | 40 (36%) | 34 (58%) *†‡* | **<0.001** |
| Body mass index (kg/m^2^) | 27 ± 6 | 22 ± 2 | 27 ± 1 *§* | 32 ± 1 *§†* | 39 ± 4 *§†‡* | **<0.001** |
| *HF characteristics* |  |  |  |  |  |  |
| HF type   - HFrEF - HFmrEF - HFpEF | 346 (60%)  100 (17%)  126 (22%) | 87 (62%)  24 (17%)  29 (21%) | 94 (64%)  27 (19%)  25 (17%) | 96 (67%)  23 (16%)  24 (17%) | 73 (51%) *‡*  22 (15%)  48 (34%) | **0.013** |
| Ischaemic heart disease | 237 (38%) | 81 (35%) | 89 (42%) | 44 (40%) | 21 (36%) | 0.5 |
| NYHA Class  I  II  III  IV | 182 (30%)  325 (52%)  100 (16%)  7 (1%) | 40 (17%)  111 (48%)  75 (32%)  4 (2%) | 31 (15%)  124 (58%)  52 (24%)  3 (1%) | 14 (13%)  59 (53%)  36 (32%)  1 (1%) | 5 (9%)  28 (48%)  23 (39%)  2 (3%) | 0.2 |
| *Comorbidities* |  |  |  |  |  |  |
| Hypertension | 216 (34%) | 61 (26%) | 72 (34%) | 53 (48%) *§* | 28 (48%) *§* | **<0.001** |
| Atrial fibrillation, history | 241 (38%) | 82 (35%) | 74 (36%) | 48 (43%) *§* | 31 (53%) *§†* | **0.038** |
| Diabetes mellitus | 138 (22%) | 32 (14%) | 41 (19%) | 36 (32%) *§†* | 28 (48%) *§†‡* | **<0.001** |
| COPD | 85 (14%) | 31 (13%) | 28 (13%) | 18 (16%) | 6 (10%) | 0.7 |
| Cardiothoracic surgery | 94 (15%) | 29 (14%) | 39 (18%) | 17 (15%) | 8 (14%) | 0.4 |
| *Laboratory tests* |  |  |  |  |  |  |
| eGFR, ml/min/1.73 m2 | 67 ± 25 | 69 ± 27 | 66 ± 24 | 64 ± 24 | 67 ± 24 | 0.4 |
| NT‐proBNP, ng/l | 1538 [558-3679] | 1820 [755-4409] | 1182 [446-2550] *§* | 1196 [450-2360] *§* | 727 [311-1702] *§* | **<0.001** |
| *Medication* |  |  |  |  |  |  |
| Beta‐blocker use | 534 (85%) | 188 (81%) | 188 (88%) | 99 (89%) | 52 (88%) | 0.078 |
| ACEi/ARB use | 521 (83%) | 192 (82%) | 183 (86%) | 96 (87%) | 45 (76%) | 0.3 |
| MRA use | 242 (39%) | 94 (40%) | 80 (37%) | 39 (35%) | 26 (44%) | 0.6 |
| Loop diuretic use | 437 (70%) | 153 (66%) | 149 (70%) | 86 (78%) | 43 (73%) | 0.2 |
| *Echocardiographic parameters* |  |  |  |  |  |  |
| LVEF (%) | 38 ± 13 | 35 ± 14 | 36 ± 13 | 40 ± 12 *§†* | 42 ± 12 *§†* | **<0.001** |
| LAVI | 43 ± 15 | 47 ± 18 | 43 ± 12 | 39 ± 11 *§* | 40 ± 13 *§* | **0.001** |
| LVMI | 107 ± 32 | 108 ± 33 | 109 ± 30 | 104 ± 34 | 105 ± 35 | 0.6 |
| e’ lateral | 8.3 ± 3.2 | 8.0 ± 3.8 | 8.0 ± 2.8 | 9.0 ± 3.2 | 9.0 ± 3.0 | **0.039** |
| e’ septal | 5.9 ± 2.3 | 5.6 ± 2.4 | 5.9 ± 2.1 | 6.3 ± 2.1 | 6.6 ± 2.7 | **0.032** |
| e’ mean | 7.1 ± 2.5 | 6.8 ± 2.7 | 7.0 ± 2.3 | 7.7 ± 2.4 | 7.8 ± 2.6 | **0.020** |
| E/e’ | 12 ± 5 | 12 ± 6 | 12 ± 5 | 12 ± 5 | 11 ± 3 | 0.4 |
| EAT | 4.8 ± 2.1 | 4.1 ± 1.7 | 4.7 ± 2.1 *§* | 5.7 ± 2.3 *§†* | 5.8 ± 1.8 *§†* | **<0.001** |
| PAT | 5.7 ± 2.8 | 4.8 ± 2.3 | 5.8 ± 2.6 *§* | 6.4 ± 3.5 *§* | 6.9 ± 3.0 *§†* | **<0.001** |

Data are mean±SD, median [interquartile range] or n (%). Final column reflects overall differences in EAT tertiles. ANOVA was used for normally distributed parameters and Chi-squared was used for categorical parameters*.
ACEi, angiotensin‐converting enzyme inhibitor; ARB, angiotensin receptor blocker; BMI, body mass index; COPD, chronic obstructive pulmonary disease; DBP, diastolic blood pressure; eGFR, estimated glomerular filtration rate; HF, heart failure; HFrEF, heart failure with reduced ejection fraction; HFmrEF, heart failure with mildly reduced ejection fraction; HFpEF, heart failure with preserved ejection fraction; LAVI, left atrial volumetric index; LVEF, left ventricular ejection fraction; LVMI, left ventricular mass index; MRA, mineralocorticoid receptor antagonist; NT‐proBNP, N‐terminal pro B‐type natriuretic peptide; NYHA, New York Heart Association; RVD, right ventricular dysfunction; SBP, systolic blood pressure. § p<.05 versus 1st quartile; † p<.05 versus 2nd quartile; ‡ p<.05 verus 3rd quartile*
